# Effect of Electromagnetic Stimulation of Amaranth Seeds of Different Initial Moisture on the Germination Parameters and Photosynthetic Pigments Content

**DOI:** 10.1038/s41598-018-32305-5

**Published:** 2018-09-19

**Authors:** Krzysztof Kornarzyński, Agata Dziwulska-Hunek, Agnieszka Kornarzyńska-Gregorowicz, Agnieszka Sujak

**Affiliations:** 1 Department of Physics, University of Life Sciences in Lublin, Akademicka 13, Lublin, Poland; 2 Department of Biophysics, University of Life Sciences in Lublin, Akademicka 13, Lublin, Poland

## Abstract

The influence of stimulation with He-Ne laser light, alternating magnetic field and the combination of these factors on germination parameters of amaranth seeds and on the chlorophyll and carotenoid content in seedlings was investigated. During the stimulation the amaranth seeds had a different initial moisture content. From the germination characteristics of the seeds as the function of humidity, three maxima and one minimum value of the germination parameters (the relative germination capacity N_*K REL*_, the relative maximum germination rate S_*K MAX REL*_ and the maximum germination index W_*K MAX*_) were obtained. In the majority of cases, the extremities coincided with the changes in the chlorophyll and carotenoid content in the seedlings. The presented research is innovative in the field of seed biology since no similar studies have been conducted before. It is difficult to interpret the results referring to the literature on this subject. The results can be explained as follows: the observed effect must be related to the stages of the water uptake by the seeds. The three stages of the water uptake associated with the seed germination process coincide with the maximum values in the germination parameters and with the change in the photosynthetic pigment content in seedlings.

## Introduction

Amaranth is a member of the *Amaranthaceae* family, genius *Amaranthus*^1^^,^ originating from South America. It is frequently referred to as the ‘grain of the future’^2–4^. Amaranth seeds have a high nutritional value, high protein content (15–18%) and preferable amino acid composition (a large amount of lysine and methionine), therefore they have become increasingly popular. Also, amaranth contains vitamins B, C, E and trace elements such as magnesium, phosphorus, iron, zinc, copper, selenium and manganese^5,6^.

The low water content in seeds results in the state of dormancy, which, in turn, results in germination inhibition^7,8^. This particular characteristic of many wild species helps them survive under stress conditions^9^. Dormancy can be induced by unfavorable environmental conditions (drought, low temperature, etc.) and occurs both in freshly harvested and mature seeds that are incapable of germination^10–12^. Seed dormancy is closely related to dehydration^7,13^.

The state of water in the caryopsis determined by NMR, represents the water contained in the air-dry seeds as an ordered system combining cyto-membrane polymers, proteins and polysaccharides with a variety of caryopsis capillaries^14–16^.

There are three distinct phases in the process of water penetration into the caryopsis^17–19^. In the first phase, the water uptake is realized via its bonding by the caryopsis biopolymers^20^. This phase is relatively short, lasts up to a few hours and is reversible. The second phase lasts up to several hours during which the hydrolysis of spare carbohydrates and proteins as well as the increase in the activity of biologically active substances take place. This phase is also reversible, but its interruption combined with the decrease in humidity reduces the vigor and vitality of the seeds. In the third phase, changes related to the onset of the seed germination process occur and they are associated with the metabolism activation. This process usually takes place at the higher level of humidity^21^. The third phase of water intake is physiologically irreversible, which means that after drying the seeds completely lose their vigor^19^.

The preparation of seeds before sowing plays a significant role in the process of germination and further development of plants. Many methods of the pre-sowing improvement of seeds such as stratification or vernalisation are used^22–26^. In order to improve the dynamics of the germination process and the uniformity of emergence, the priming method consisting of hydrating of the seeds under strictly controlled conditions and then drying them in order to obtain their initial water content is applied^27–29^.

Physical factors used in the pre-sowing seed stimulation include static and alternating magnetic and electric fields, ionizing radiation, microwave and laser radiation^30–33^ as well as magnetically treated water^34–36^. Positive effects on germination capacity, plant growth and improvement of yields of electromagnetic stimulation (ELM) of seeds have been confirmed by many researchers^37–39^. The interaction of electromagnetic fields with living organisms affects the permeability of ion channels in biological membranes^38,40^ and it also influences biological functions of organisms by altering hormone concentrations and inducing changes in DNA synthesis or transfer^41,42^. In addition, the stimulation with ELM influences many physiological and biochemical processes in plant cells. Some experiments have shown that the stimulation with magnetic field can increase grouping of the protein and minerals, improve enzyme activity^41^ or photosynthesis^43^ and biological membrane integrity^39^.

For example, the ion cyclotron resonance (ICR) of the Ca^2+^ ion subjected to weak magnetic fields (such as the Earth’s magnetic field) of low frequencies may affect germination. Ca^2+^ ions, which participate in the regulation of plant growth and development, influence photomorphology, embryogenesis, gravitation and phototropism at all stages of the seed germination and plant growth^44^. In addition, studies on plants have shown that weak magnetic fields can cause Ca^2+^ equilibrium disturbances and different biochemical changes^45–47^.

Vashisth and Nagarajan^48^ found that the stimulation with alternating magnetic field led to an increase in the length of the sunflower roots. The application of an impulse magnetic field of 35–80 J/impulse to the cotton seed stimulation resulted in an increase in the water transpiration rate, photosynthesis yield, stomatal conductance, root length increase, shoot height and N, P, K, Ca and Mg content at the early stages of growth^49^. Radhakrishnan *et al*.^50^ applied the 10 Hz alternating magnetic field to stimulate soybean seeds. The significant increase in Fe, Cu, Mn, Zn, Mg, K and Na content (yet, at the same time, the lower Ca content), the increased protein content and the increased activity of various enzymes in the stimulated seedlings were observed.

Mroczek-Zdyrska *et al*.^51^ conducted research in which, during a fourteen-day vegetation period, sprouting and growing lupine (*Lupinus angustifolius* L.) seeds were exposed to the constant magnetic field with an induction of 130 mT. A faster growth of shoots was observed in the terrestrial part. When compared to control (untreated) plants, the seedlings showed an increased chlorophyll a/b ratio and a visible decrease in the ratio of total chlorophyll (*a* + *b*)/*C* to carotenoid. Other studies examined the influence of the alternating magnetic field of 0.2 mT induction and a frequency of 16 Hz and 50 Hz on lupine seeds during 14 days of constant field exposure. In both cases, the alternating magnetic field reduced the chlorophyll and carotenoid content in leaves^52^.

The usage of a static magnetic field to stimulate soybean seeds by Shine *et al*.^53^ resulted in the increased reactive oxygen species (ROS) production in the germinated seeds. The stimulation of pea seeds with a constant magnetic field (125 mT and 250 mT) produced a positive effect on the initial growth of seedlings^54^. Chickpea seeds (*Cicer arietinum* L.) stimulated with a 250 mT constant magnetic field responded with a significant increase in germination rates, seedlings and young plants length as well as in the increase of the dry weight of one-month old plants^55^.

A constant 0.15 T magnetic field with the exposure times of 0 (control), 3, 6, 9 and 12 minutes was used to stimulate lentil seeds (*Lens Culinaris*, Med.)^33^. Before the stimulation took place, the seeds had been immersed in distilled water for 1 h. It was assumed that the intracellular water, because of its magnetic properties, plays a role in the absorption of magnetic field energy. Data analysis showed that germination energy and germination percentage reached the highest values for unstimulated samples. The longest stems and roots were found in plants subjected to a magnetic field between 6 and 9 minutes. Similar positive effects of stimulation were observed for the total weight and the above-mentioned stimulation times. The longer exposure time of 12 minutes resulted in lower germination parameters than the parameters achieved during the exposure time of 3 minutes^33^.

Hernandez *et al*.^37^ reported that the most likely explanation for the stimulation of seeds is its influence on the physiological and biochemical processes in seeds taking place as a result of the transformation of light energy into chemical energy. On the other hand, Vasilevski^24^ stated that the influence of laser light on living organisms is based on the increase in energy equilibration resulting from the transformation of light energy into other energy sources as electric (increasing the energetic stability of the cell) and by increasing of the electrical potential of bio membranes in plant cells. The increase in the activity of amylolytic enzymes, the increase in dry matter content of roots, leaves and stems, and the increase of free radicals in seedlings after five-fold exposure to a He-Ne laser radiation^56,57^ were obtained for white faba bean and white lupine seeds. The experiments on He-Ne laser stimulation of scorzonera seeds conducted on Petri dishes showed an increase in the seed germination capacity and, in a field experiment, the increase of seedlings length and chlorophyll and carotenoid content for most of the applied doses was observed^58^. Ćwintal *et al*.^59^ stimulated *alfalfa* seeds with He-Ne laser light and obtained a significant increase in the protein, P and Mo content, and a decrease in the crude fibre content relative to the dry weight of the plants.

Perveen *et al*.^60^ soaked sunflower seeds in water for 3 h and subjected them to the He-Ne laser beam of ϕ = 1.5 mm to obtain a significant increase in the shoot length, fresh and dry root mass and their diameter, leaf numbers per plant and K, Ca and Mg content.

A He-Ne impulse laser (the wavelength of 632.8 nm, the power of 100 W m^−2^, the pulse duration of 1 min.) was used to stimulate carrot seeds (*Daucus carrota* L., cv. Nantes)^33^. The 5-, 7- and 9-fold laser irradiation was applied. The best results concerning germination energy were obtained in the case of the 5-fold irradiation, while in the case of germination percentage 7-fold treatment was used. On the other hand, the 9-fold treatment showed the germination inhibitory effect. The influence of the pre-sowing laser stimulation on beans (*Phaseolus vulgaris* L., cv. Plovdiv) soaked in water before the treatment was also tested using a high power (176 W · m^−2^) He-Ne laser with the exposure times of 0 min (control), 5, 10 and 15 minutes^33^. A slightly positive effect of stimulation was obtained for a five-minute exposure time, while higher doses resulted in the negative effects as compared to the control group. The exceptionally negative effect of the stimulation was related to the longest applied exposure time of 15 minutes. In that case all of the tested germination and growth parameters were several times lower as compared to that obtained for control group. The authors presumed that the combination of high light intensity with an exposure time longer than 5 minutes probably introduced too much energy into the cell, which lead to the inhibition of the germination process and plant growth^33^.

Chlorophylls and carotenoids participate in the transport of light energy to the reaction centers of photosynthetic proteins^61^. In higher plants, mainly chlorophylls *a* and *b* are found and their approximate ratio amounts to 3:1^62^. Sun-exposed plants reach a relatively high photosynthesis yield as well as high concentrations of chlorophylls^62^. In shade-loving plants, the ratio of chlorophyll *a* to *b* ranges between 2.0–2.8, while in sun-loving plants it ranges between 3.5–4.9^61,63,64^.

The purpose of the study was to determine the influence of He-Ne laser light, alternating magnetic field and the combination of these factors on the amaranth seed germination kinetics and on the content of chlorophyll and carotenoids in seedlings. During the stimulation with physical factors, the amaranth seeds had a different initial humidity. An attempt was made to explain the impact of the electromagnetic stimulation of the seeds on seed germination assuming that the observed effect is related to the consecutive stages of the water uptake by seeds.

## Results

**Figure 1:**
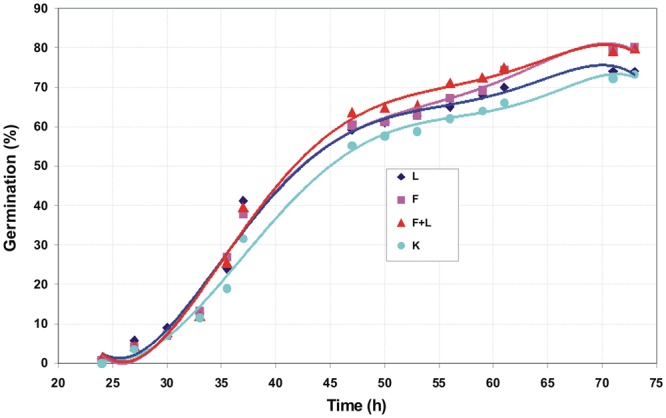
Course of germination of amaranth seeds for initial moisture content W = 7.78%.

**Figure 2:**
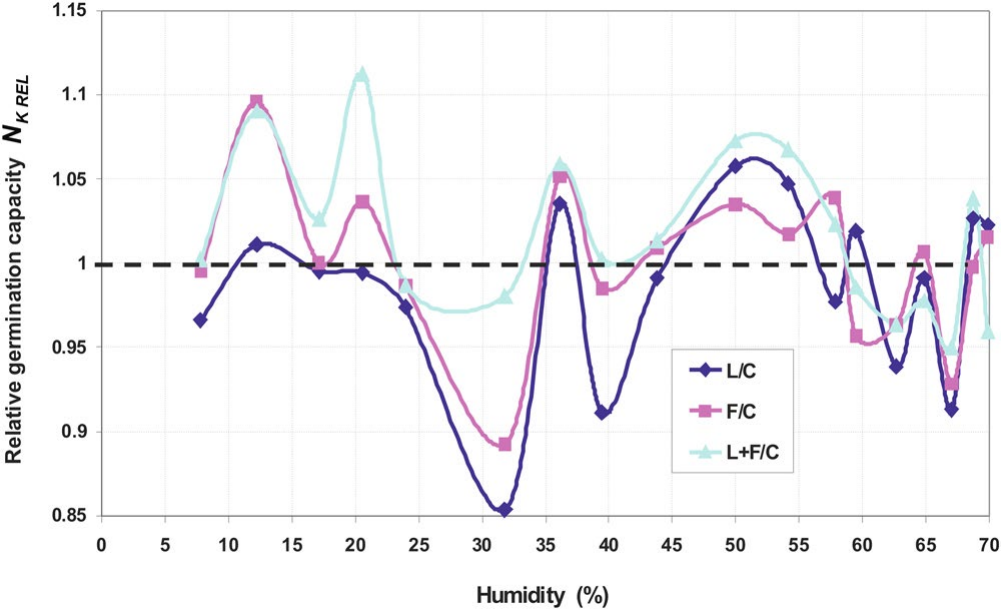
Relative germination capacity as a function of humidity during stimulation.

**Figure 3:**
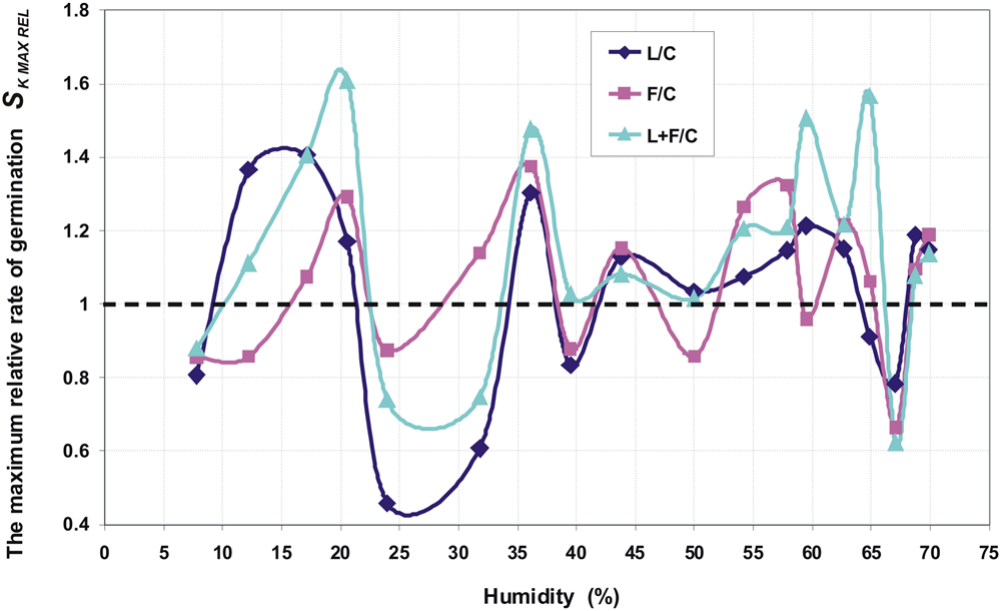
The maximum relative rate of germination of amaranth seeds as function of humidity during stimulation.

**Figure 4:**
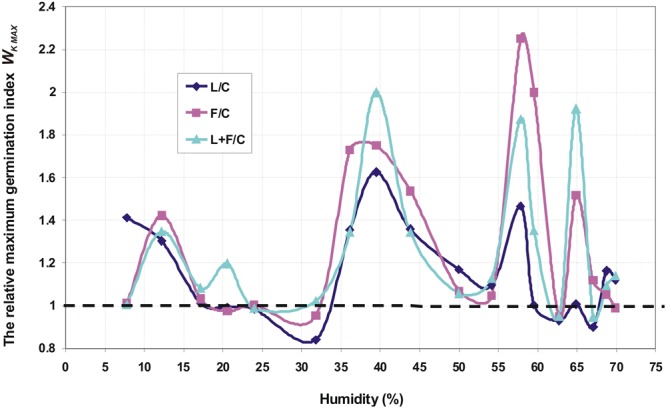
The relative maximum germination index for amaranth seeds as function of humidity during stimulation.

Figure 1 shows an exemplary germination kinetics of amaranth seeds. Figures 2, 3 and 4 show the relationship of parameters characterizing the germination of amaranth seeds as a function of their humidity during stimulation. Indicated humidity is that at which the seeds were subjected to stimulation. The relative germination capacity *N*_*K REL*_ is the ratio of the total germination capacity of the L, F and L + F stimulated sample (L -stimulation with a He-Ne laser with a surface power density of 3 mW · cm^−2^ over 10 s, F - stimulation with an alternating magnetic field of the frequency of 50 Hz, magnetic induction 30 mT over 30 s, L + F - stimulation with both factors combined) to control sample (C) for the given seed moisture during stimulation. The value of the maximum relative germination index *W*_*K MAX*_ was determined from the seed germination data (as a function of time) as the maximum relative value for a particular seed moisture during the stimulation with L, F or L + F. The maximum relative germination rate *S*_*K MAX REL*_ was determined from the measured data (the germination rate versus time) as a maximum value for the L, F or L + F stimulated sample. All the germination parameters were determined for a mean of five measurements. Three maximal values for the relative germination capacity *N*_*K REL*_, the relative germination rate *S*_*K MAX REL*_ and the maximum (relative) germination index *W*_*K MAX*_ can be distinguished. The first maximum value is within the range of humidity ΔW_I_ = 10–20% and lasts from 0.3 (0.5) to 2 hours of water absorption by the seeds placed on the blotting paper – as read from the water intake chart; the second ΔW_II_ = 35–45% lasting from 3.5 to 6.5 hours, and the third maximum ΔW_III_ = 55–65% - from 10^th^ to 18^th^ hour of water intake. A clear minimum value for all the above germination parameters is also visible in the seed moisture content ΔW_min_ = 25–35%. In the case of the relative germination capacity *ΔN*_*K RELL*_ (Fig. 2), the maximum value ΔW_I_ max for the laser stimulation is not observed, and in the range of 20–35% of humidity the minimum value appears representing the negative impact of stimulation on seed germination. For all other types of stimulation all maxima overlap.

Table 1 shows the content of chlorophyll *a* in the amaranth seedlings. In all experimental variants, the presence of the local maxima and minima of chlorophyll *a* concentrations can be observed. For control, local chlorophyll *a* concentration maxima are present at humidity of 17.2%: 684.75 μg · g^−1^ and 43.81%: 377.77 μg · g^−1^ and minimum at 36.13%: 204.36 μg · g^−1^. The highest chlorophyll *a* concentration was found for all the experimental variants at 17.2% humidity: L - 608.15 μg · g^−1^, F - 546.91 μg · g^−1^, L + F - 742.10 μg · g^−1^. Minimal chlorophyll contents were found for similar humidity: L - 167.93 μg · g^−1^ for 49.98% humidity, F - 172.28 μg · g^−1^ for humidity of 43.81% and L + F - 160.86 μg · g^−1^ for humidity of 49.98%.

**Table 1:** Concentrations of chlorophyll *a* in amaranth seedlings.

Seed humidity (%)	Chlorophyll *a* (µg · g^−1^)			
	C	L	F	L + F
7.78	359.13^aA^ ± 7.49	239.83^bA^ ± 0.97	311.94^bA^ ± 3.28	347.49^bA^ ± 2.58
12.19	267.66^aB^ ± 3.04	196.41^bB^ ± 3.83	312.97^bA^ ± 5.79	142.54^bB^ ± 8.64
17.12	684.75^aB^ ± 12.08	608.15^bB^ ± 7.57	546.91^bB^ ± 17.62	742.10^bB^ ± 11.75
20.54	267.66^aB^ ± 3.04	144.03^bB^ ± 7.14	222.63^bB^ ± 11.99	190.30^bB^ ± 3.88
23.95	264.02^aB^ ± 1.72	200.50^bB^ ± 4.32	324.45^bB^ ± 4.12	311.39^bB^ ± 2 0.17
31.79	267.66^aB^ ± 3.04	168.06^bB^ ± 6.11	190.07^bB^ ± 15.24	139.87^bB^ ± 5.44
36.13	204.36^aB^ ± 6.14	239.55^bA^ ± 4.88	217.87^bB^ ± 6.11	236.64^bB^ ± 3.75
39.47	267.66^aB^ ± 3.04	153.29^bB^ ± 4.70	184.58^bB^ ± 2.53	205.09^bB^ ± 4.52
43.81	377.77^aB^ ± 4.21	317.03^bB^ ± 2.25	172.28^bB^ ± 1.94	316.93^bB^ ± 3.87
49.98	325.35^aB^ ± 6.18	167.93^bB^ ± 1.65	206.40^bB^ ± 2.78	160.86^bB^ ± 0.94
54.16	272.23^aB^ ± 9.94	256.31^bB^ ± 5.29	289.55^bB^ ± 1.91	274.10^aB^ ± 1.13
57.84	272.23^aB^ ± 9.94	213.06^bB^ ± 4.85	236.98^bB^ ± 2.06	230.43^bB^ ± 1.29
59.51	272.23^aB^ ± 9.94	260.20^bA^ ± 2.16	243.67^bB^ ± 7.42	311.79^bB^ ± 1.27
62.69	272.23^aB^ ± 9.94	205.72^bB^ ± 1.07	239.28^bB^ ± 2.18	239.57^bB^ ± 1.72
64.87	344.55^aB^ ± 9.92	384.99^bB^ ± 1.49	402.74^bB^ ± 3.99	303.48^bB^ ± 3.71
67.04	344.55^aB^ ± 9.92	261.64^bB^ ± 1.74	197.43^bB^ ± 2.73	494.62^bB^ ± 3.46
68.72	344.55^aB^ ± 9.92	351.30^aA^ ± 2.16	392.60^bB^ ± 2.91	707.94^bB^ ± 2.86
69.89	344.55^aB^ ± 9.92	876.02^bB^ ± 3.85	306.95^bA^ ± 14.53	476.23^bB^ ± 8.75

C – control, L -stimulation with a He-Ne laser light, F - stimulation with an alternating magnetic field, L + F - stimulation with both combined factors.

For chlorophyll *b* content (Table 2), in all stimulation scenarios, the maximum concentration was observed for 17.2% humidity (C: 175.83 μg · g^−1^, L: 161.00 μg · g^−1^, F: 142.61 μg · g^−1^, L + F: 198.46 μg · g^−1^). The minimum values are found in the range of humidity between 23.95% and 62.69%.

**Table 2:** Concentrations of chlorophyll *b* in amaranth seedlings.

Seed humidity (%)	Chlorophyll *b* (µg · g^−1^)			
	C	L	F	L + F
7.78	97.32^aA^ ± 3.05	66.69^bA^ ± 0.82	79.17^bA^ ± 1.04	86.42^bA^ ± 1.79
12.19	81.52^aB^ ± 3.31	68.26^bA^ ± 5.01	97.17^bB^ ± 3.10	56.89^bB^ ± 4.75
17.12	175.83^aB^ ± 4.12	161.00^bB^ ± 5.33	142.61^bB^ ± 4.05	198.46^bB^ ± 2.31
20.54	81.52^aB^ ± 3.31	58.34^bB^ ± 5.34	83.59^aA^ ± 3.90	72.24^bB^ ± 2.03
23.95	70.17^aB^ ± 1.20	54.13^bB^ ± 0.98	100.59^bB^ ± 23.15	84.02^aA^ ± 6.10
31.79	81.52^aB^ ± 3.31	64.57^bA^ ± 0.66	77.90^aA^ ± 5.10	57.57^bB^ ± 3.37
36.13	57.86^aB^ ± 1.14	66.07^bA^ ± 0.88	57.67^aB^ ± 3.02	62.66^bB^ ± 1.55
39.47	81.52^aB^ ± 3.31	58.30^bB^ ± 2.58	72.30^bB^ ± 1.69	70.60^bB^ ± 1.05
43.81	101.48^aA^ ± 2.86	87.24^bB^ ± 1.21	45.69^bB^ ± 0.33	87.95^bA^ ± 0,97
49.98	86.88^aB^ ± 4.47	44.53^bB^ ± 2.45	55.03^bB^ ± 0.11	41.38^bB^ ± 1.70
54.16	71.71^aB^ ± 2.69	68.92^aA^ ± 1.63	82.92^bA^ ± 2.68	74.94^aA^ ± 2.48
57.84	71.71^aB^ ± 2.69	57.09^bB^ ± 0.79	67.80^aB^ ± 3.01	65.29^bB^ ± 1.89
59.51	71.71^aB^ ± 2.69	69.74^aB^ ± 0.91	68.36^aB^ ± 3.23	87.75^bA^ ± 3.14
62.69	71.71^aB^ ± 2.69	55.49^bB^ ± 0.89	65.29^bB^ ± 2.11	66.99^bB^ ± 1.09
64.87	88.60^aB^ ± 4.20	101.11^bB^ ± 0.18	108.64^bB^ ± 4.27	85.84^aA^ ± 3.76
67.04	88.60^aB^ ± 4.20	80.87^aB^ ± 4.24	62.76^bB^ ± 8.01	149.19^bB^ ± 1.39
68.72	88.60^aB^ ± 4.20	101.17^bB^ ± 2.22	110.88^bB^ ± 3.24	195.70^bB^ ± 0.71
69.89	88.60^aB^ ± 4.20	232.21^bB^ ± 5.24	86.81^aB^ ± 3.84	137.14^bB^ ± 3.27

Table 3 shows the ratio of chlorophyll *a* to chlorophyll *b*, which ranges between 2.88–4.02 with the mean values of 3.67, 3.44, 3.41 and 3.42, respectively, for C, L, F and L + F sample. For the moisture content of 20.54% and 31.79%, a decrease in chlorophyll *a* to *b* ratio was observed in the stimulated samples compared to the values obtained for control sample (the value below 3). Table 4 shows the content of carotenoids in the amaranth seedlings. Local maxima and minima are observed and they are, in most cases, correlated with a change in the chlorophyll *a* concentration. In most cases, the ratio of the total chlorophylls to the carotenoid content was present in the range between 4.2 to 6.12 (Table 5). For the moisture content of 36.13 and 49.98, there are a number of local minima where the ratio of chlorophyll *a* and *b* to total carotenoids (a + b)/C was below 4. These values are correlated with the occurrence of minima in the chlorophyll *a* content.

**Table 3:** Chlorophyll *a/b* ratio in amaranth seedlings.

Seed humidity (%)	Chlorophyll *a*/Chlorophyll *b*			
	C	L	F	L + F
7.78	3.69^aA^	3.60^aA^	3.94^aA^	4.02^aA^
12.19	3.28^aA^	2.88^aB^	3.22^aB^	2.51^bB^
17.12	3.89^aA^	3.78^aA^	3.84^aA^	3.74^aA^
20.54	3.28^aA^	2.47^bB^	2.66^bB^	2.63^bB^
23.95	3.76^aA^	3.70^aA^	3.23^aB^	3.71^aA^
31.79	3.28^aA^	2.60^bB^	2.44^bB^	2.43^bB^
36.13	3.53^aA^	3.63^aA^	3.78^aA^	3.78^aA^
39.47	3.28^aB^	2.63^bB^	2.55^bB^	2.90^bB^
43.81	3.72^aA^	3.63^aA^	3.77^aA^	3.60^aA^
49.98	3.74^aA^	3.77^aA^	3.75^aA^	3.89^aA^
54.16	3.80^aA^	3.72^aA^	3.49^aA^	3.66^aA^
57.84	3.80^aA^	3.73^aA^	3.5^aA^	3.53^aA^
59.51	3.80^aA^	3.73^aA^	3.56^aA^	3.55^aA^
62.69	3.80^aA^	3.71^aA^	3.66^aA^	3.58^aA^
64.87	3.89^aA^	3.81^aA^	3.71^aA^	3.54^aA^
67.04	3.89^aA^	3.24^aA^	3.15^aB^	3.32^bB^
68.72	3.89^aA^	3.47^aA^	3.54^aA^	3.62^aA^
69.89	3.89^aA^	3.77^aA^	3.54^aA^	3.47^aB^

**Table 4:** Carotenoid concentrations in amaranth seedlings.

Seed humidity %	Carotenoids (µg · g^−1^)			
	C	L	F	L + F
7.78	94.61^aA^ ± 2.30	74.71^bA^ ± 1.36	87.60^bA^ ± 1.98	95.23^aA^ ± 2.23
12.19	77.98^aB^ ± 8.68	60.85^aB^ ± 3.12	57.71^aB^ ± 23.49	40.79^bB^ ± 6.66
17.12	169.83^aB^ ± 7.64	148.97^aB^ ± 5.70	166.85^aB^ ± 30.91	187.69^aB^ ± 14.42
20.54	77.98^aB^ ± 8.68	40.01^bB^ ± 6.14	55.83^bB^ ± 13.72	53.29^bB^ ± 7.52
23.95	75.13^aB^ ± 5.44	58.72^bB^ ± 5.86	89.96^bA^ ± 11.24	105.62^bA^ ± 4.67
31.79	77.98^aB^ ± 8.68	45.36^bB^ ± 9.46	51.14^bB^ ± 3.63	40.76^bB^ ± 3.63
36.13	73.09^aB^ ± 4.57	82.68^bA^ ± 3.38	77.02^aA^ ± 3.67	85.90^bA^ ± 2.95
39.47	77.98^aB^ ± 8.68	41.14^bB^ ± 3.61	41.95^bB^ ± 8.86	43.03^bB^ ± 7.24
43.81	96.50^aA^ ± 1.25	87.84^bB^ ± 0.55	62.78^bB^ ± 1.02	93.32^bA^ ± 1.44
49.98	108.53^aB^ ± 5.61	62.58^bB^ ± 3.58	76.45^bA^ ± 7.83	53.12^bB^ ± 0.40
54.16	64.53^aB^ ± 4.06	64.17^aA^ ± 4.64	66.90^aB^ ± 2.77	66.76^aB^ ± 3.75
57.84	64.53^aB^ ± 4.06	52.09^bB^ ± 0.58	60.69^aB^ ± 1.21	55.09^bB^ ± 4.61
59.51	64.53^aB^ ± 4.06	61.38^aB^ ± 1.36	52.76^bB^ ± 3.44	68.12^aB^ ± 1.39
62.69	64.53^aB^ ± 4.06	45.95^bB^ ± 3.32	54.65^bB^ ± 2.57	63.75^aB^ ± 2.06
64.87	70.81^aB^ ± 3.00	80.92^bA^ ± 2.34	94.46^bA^ ± 2.66	67.03^aB^ ± 2.27
67.04	70.81^aB^ ± 3.00	95.93^bB^ ± 2.51	59.38^bB^ ± 3.18	160.84^bB^ ± 5.25
68.72	70.81^aB^ ± 3.00	85.41^bA^ ± 3.50	93.30^bA^ ± 1.94	156.76^bB^ ± 5.00
69.89	70.81^aB^ ± 3.00	202.69^bB^ ± 1.14	66.88^aA^ ± 5.88	108.53^bB^ ± 2.34

**Table 5:** Ratio of sum of the chlorophylls (*a + b*) to carotenoids.

Seed humidity (%)	Chlorophyll (*a* + *b*)/Carotenoids			
	C	L	F	L + F
7.78	4.82^aA^	4.10^bA^	4.46^aA^	4.56^aA^
12.19	4.48^aA^	4.35^aA^	7.11b^B^	4.89^aB^
17.12	5.07^aA^	5.16^aB^	4.13^bA^	5.01^aA^
20.54	4.48^aB^	5.06^aB^	5.48^bB^	4.93^aA^
23.95	4.45^aA^	4.34^aA^	4.72^aA^	3.74^bB^
31.79	4.48^aB^	5.13^bB^	5.24^bB^	4.84^aA^
36.13	3.59^aB^	3.70^aA^	3.58^aA^	3.48^aB^
39.47	4.48^aA^	5.14^bB^	6.12^bB^	6.41^bB^
43.81	4.97^aA^	4.60^aB^	3.47^bB^	4.34^aA^
49.98	3.8^aB^	3.40^aB^	3.42^aB^	3.81^aB^
54.16	5.33^aA^	5.07^aB^	5.57^aB^	5.23^aB^
57.84	5.33^aA^	5.19^aB^	5.02^aA^	5.37^aB^
59.51	5.33^aA^	5.38^aB^	5.91^bB^	5.87^aB^
62.69	5.33^aA^	5.68^aB^	5.57^aB^	4.81^bA^
64.87	6.12^aB^	6.01^aB^	5.41^bB^	5.81^aB^
67.04	6.12a^B^	3.57b^A^	4.38b^A^	4.00^bA^
68.72	6.12^aB^	5.30^bB^	5.40^bB^	5.76^aB^
69.89	6.12^aB^	5.47^bB^	5.89^aB^	5.65^aB^

## Discussion

The paper presents the effect of pre-sowing electromagnetic stimulation of seeds with a certain initial moisture content. Both positive and negative effects on the germination parameters of the seeds relative to the control values were observed and they depended on the moisture content during stimulation (Figs 2–4). Amaranth seeds with a different initial moisture content during stimulation were in different phases of the germination process. It is difficult to interpret the results on the basis of the literature research because such studies have not been conducted before. In our studies, the obtained germination parameters and the resulting maximal and minimal values relative to the control sample are indicative of the effects of the various electromagnetic factors on the germination process.

Similarly to the studies presented here, many authors examined the effect of a He-Ne laser and alternating magnetic field on the germination of amaranth seeds. Dziwulska-Hunek *et al*.^2^ used the laser beam stimulation of the power density of 6 mW/cm^2^, the alternating magnetic field with the induction 30 mT, the frequency 50 Hz for t = 30 s as well as the combination of both factors for amaranth seeds cvs Aztek and Rawa. The effects on the germination of the seeds were examined on Petri dishes in darkness at temperatures from 10 to 55 °C. The most pronounced effects important for the germination process were registered at the temperatures of 20 and 25 °C^2^. When the above parameters of the stimulation of amaranth seeds cv. Rawa were present, the increase in dry matter, raw protein, crude fiber, crude ash and in the final yield was observed. An increase in the levels of Leu, Val, Lys, and Phe + Tyr amino acids as well as decreased levels of Arg, Glu and Ala. The levels of Cys, Thr, Ile, His and Pro remained unchanged^3^.

Sujak and Dziwulska-Hunek^65^ applied a laser beam of the power density of 6 mW/cm^2^, alternating magnetic field with an induction of B = 30 mT and frequency of 50 Hz for t = 30 s, and the combination of these factors to stimulate amaranth seeds. A significant decrease in the levels of K, Mg, Ca, Na, Cu and Mn as well as a significant increase in Zn and two-fold increase of the level of Fe was observed as compared to the control sample. Electromagnetic stimulation resulted in an increase in essential fatty acids and a decrease in most of saturated fatty acids, which is beneficial for healthy eating. Using the stimulating factors (L, F and L + F) at the above-mentioned doses, Dziwulska-Hunek *et al*.^4^ stimulated the seeds of amaranth, cv. Rawa, and subjected them to germination on Petri dishes, in pots and in field trials. As a result, the germination capacity and energy increased for all the stimulated seeds for the seed sown on the Petri dishes but a significant reduction for combined stimulation factors (L + F) was observed in the field tests. There was no effect on the carotenoids and chlorophyll content in all experimental layouts.

In some studies, before the stimulation with ELM factors took place, seeds had been soaked in distilled water. It was assumed that intracellular water takes part in magnetic field energy absorption due to its magnetic properties. Such an explanation was given concerning a positive effect of magnetic field simulation on soybeans^33^. When the soybeans had been soaked before they were stimulated with a strong He-Ne laser, negative effects were observed and, in some cases, a strong germination- and growth-slowing down effect^33^.

In the water-soaked seeds part of the light energy must be absorbed by the incorporated water, which is why chlorophyll centres receive less energy compared to the dry seeds samples. For both of the above-mentioned cases, the seed moisture content before stimulation had not been determined.

**Figure 5:**
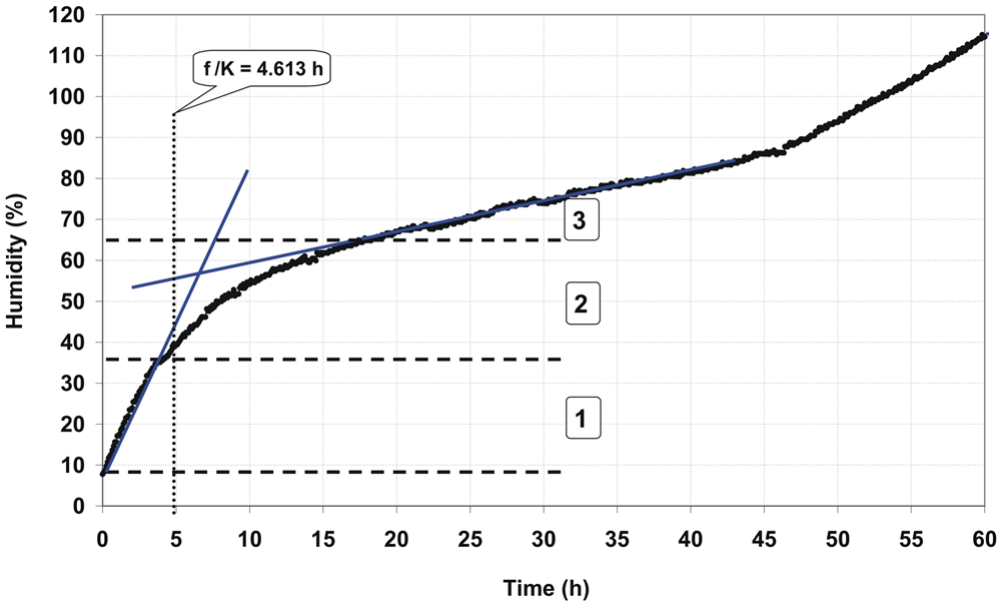
Kinetics of water uptake by amaranth seeds in a capillary-weight system.

In our studies, stimulation was performed on moist (hydrated) seeds, at the moment when no sprouts had yet appeared. The first sprouts appeared 23–25 hours after the seeds had been planted on tissue paper. Stimulation with ELM factors may have an impact on the processes that take place in seeds at different stages of water uptake. It is not known when, for particular phases of water intake, the process is the fastest and whether in our case it coincides with the moment of stimulation. Assuming that the three stages of water uptake are related to the maximum values of the germination parameters (values of *N*_*K REL*_, *S*_*K MAX REL*_ and *W*_*K MAX*_ - Figs 2–4), we can explain our results by the effects of stimulation on seeds of different humidity during stimulation. The three phases of water uptake can be explained by the examination of the water uptake curve (Fig. 5).

Assuming that the phases of water uptake and the processes occurring in the seeds are affected by the stimulating factors, the appearance of three maxima for the germination parameters can be combined with the three phases of water uptake during the early stages of the germination process. The first phase (I) lasts until the end of the rectilinear portion of the line tangent to the water uptake graph, while the third phase (III) starts from the end of rectilinear portion of the second line which is tangent to the graph – Fig. 5. As seen at the graphs 4–6, these phases overlap with the maximal values of the germination parameters where the first one appears in the moisture range ΔW_I_ = 10–20% and takes from 0.3 (0.5) to 2 hours of water absorption by the seeds placed on the blotting paper - read from the water uptake graph, the second one: ΔW_II_ = 35–45% takes between 3.5 and 6.5 hours and the third maximum ΔW_III_ = 55–65% from 10 to 18 hours of water uptake.

Application of the logistic model for curve fitting to the measuring points: *Z*_*N*_ = *Z*_*K*_ (1 + *exp*(*β* *−* *K* · *t*))^*−*1^, where: *Z*_*N*_ - water content at time *t*, *Z*_*K*_ - final water content in the seeds before germination (*Z*_*K*_ = 70.49), β (1.024) and *K* (0.222) – coefficients and *t* - time, allows for determination of the inflection point of the logistic curve, where *β/K* is the time to reach the inflection point of the logistic curve. It is noteworthy that the logistic curve is symmetric concerning its inflection point^66^. The time taken to obtain the logistic curve inflection point *t*_*βk*_ = *β/K* amounts to 4.613 hours and can be considered as the beginning of the second phase of the germination process - Fig. 5.

Concentrations of chlorophylls *a* and *b* were similar to those previously published for amaranth^4^. In our case, the chlorophyll content varied significantly depending on the kind of the applied stimulation and the seed moisture at which it occurred. It is interesting that previous studies conducted on dry seedlings indicated no significant changes in the chlorophyll content under stimulation with electromagnetic factors^4^.

The weight ratio of chlorophyll *a* and chlorophyll *b* (*a/b* ratio) is an indicator of the functional pigment equipment and light adaptation/acclimation of the photosynthetic apparatus. Chlorophyll *b* is found exclusively in the pigment antenna system, whereas chlorophyll *a* is present in the reaction centres of photosystem I and II as well as in the pigment antenna^67^. While the light-harvesting pigment protein LHC-I of the photosynthetic pigment system PSI has an *a/b* ratio of ~3, the ratio of LHC-II of PS II is variable and shows a light adaptation/acclimation response. Shade plants possess much higher amounts of LHC-II than sun-exposed plants and, consequently, their *a/b* ratios are lower than in sun-exposed plants. Thus, a decrease in the chlorophyll *a/b* ratio may be interpreted as an enlargement of the antenna system of PS II.

Lupine seeds germinated and grew in a constant magnetic field of an induction of 130 mT. Faster shoot growth in the aerial part of plants was achieved accompanied by the increase in chlorophyll a/b ratio (by 37%) and a decrease in the ratio of the sum of the chlorophylls to carotenoids (a + b)/C in the seedlings as compared to control parameters. The ratio of chlorophyll a/b is known as an indirect energy activity indicator of LHC II. LHC II controls the first stage of conversion of solar energy to chemical energy. That observation was consistent with the results reported by Racuciu *et al*.^68^ for maize where a slight increase (3%) of this ratio at the low magnetic induction of 50 mT after 14 days of growth was observed. Interestingly for the magnetic field with the induction of 100 mT, the chlorophyll content decreased by 4%.

Pazur *et al*.^69^ demonstrated that a weak magnetic field lowered the content of protochlorophyll pigment (PChlide) and carotenoids in barley seedlings (*Hordeum vulgare*).

Similarly, longer exposition to magnetic field reduced the level of assimilation pigments in *Zea mays* L.^68^ and acacia robinia (*Robinia pseudoacacia* L.)^70^.

Studies on the effect of scorzonera seed stimulation with a He-Ne light on germination and chlorophylls and carotenoid contents were conducted for seeds with different initial germination abilities. Increases in the chlorophyll and carotenoids content in 8-day seedlings sown on Petri dishes were observed as well as in 30-day seedlings in field experiments^58^. Lucerne and lupine seeds were stimulated with laser light, alternating magnetic field, and a combination of both factors. As a result, an increase in chlorophyll *a* and *b* concentrations in seedlings of both species was observed^4^. In the case of stimulation with magnetic field and with laser light, exposure time had no effect on germination parameters and content of chlorophylls in the soy seedlings. The stimulation with laser light had less effect than magnetic field^2^.

In our case seedlings from all experimental variants were grown under the same lighting conditions, so the differences observed here must be the effect of the seed stimulation at a certain humidity value. It is difficult to interpret the results because the ratio of chlorophyll *a* to chlorophyll *b* (Table 5) in most cases was within the range of values obtained by other authors, assuming the correct method of isolation of the photosynthetic pigments with use of acetone^71,72^. However, for the 20.54% and 31.79% of humidity, the decrease in the chlorophyll *a* to *b* ratio in the stimulated samples (below 3) was observed as compared to control parameters.

In order to interpret properly the results concerning the chlorophyll *a* and *b* contents in plants, it is necessary to use strictly defined controls. It is recommended to use the mass of chlorophyll *a* and *b* per m^2^ of leaf area. Because in our case the chlorophyll content was measured on seedlings, some of which had fairly developed leaves and some did not, it is difficult to estimate their absolute value and compare them correctly. It is permissible to use the chlorophyll content on the dry sample mass or the wet sample mass. The latter was used in our case. Unfortunately, different water content in the wet mass can cause interpretation problems. For example, the increase in chlorophyll content per unit of fresh weight may be due to the decrease in fresh weight relative to control due to loss of water caused by some factors^67^.

The weight ratio of chlorophyll *a* and *b* to total carotenoids (*a* + *b*)*/C* is an indicator of the greenness of plants. The ratio (*a* + *b*)*/C* normally lies between 4.2 and 5 in sun leaves and sun-exposed plants, and between 5.5 and 7.0 in shade leaves and shade-exposed plants. Lower values of the ratio (*a* + *b*)*/C* are an indicator of senescence, stress and damage to the plant and the photosynthetic apparatus expressed by faster breakdown of chlorophylls than carotenoids^72,73^.

In most cases, the ratio of chlorophyll content to carotenoids was in the range between 4.2 and 6.12 (Table 5). For humidity 36.13 and 49.98, there are a number of local minima where the ratio of chlorophyll *a* and *b* to total carotenoids (*a* + *b*)*/C* is smaller than 4. These values are correlated with the occurrence of minima in the chlorophyll *a* content which is placed within PSII protein and takes part in photosynthesis. It is possible that this result gives information about some type of stress on the photosynthetic apparatus at an early stage of seedling development - at ambient humidity. It is possible that during the absorption of water, a certain type of stress on the cells results in a change of the nature of its intake, which results in a change in the content of enzymes responsible for the synthesis of photosynthetic pigments at a later stage.

## Methods

Amaranth seeds cv Rawa were sown on Petri dishes lined with three layers of filter (blotting) paper moistened with distilled water. After placement on moist paper the seeds took water to raise their moisture.

**Figure 6:**
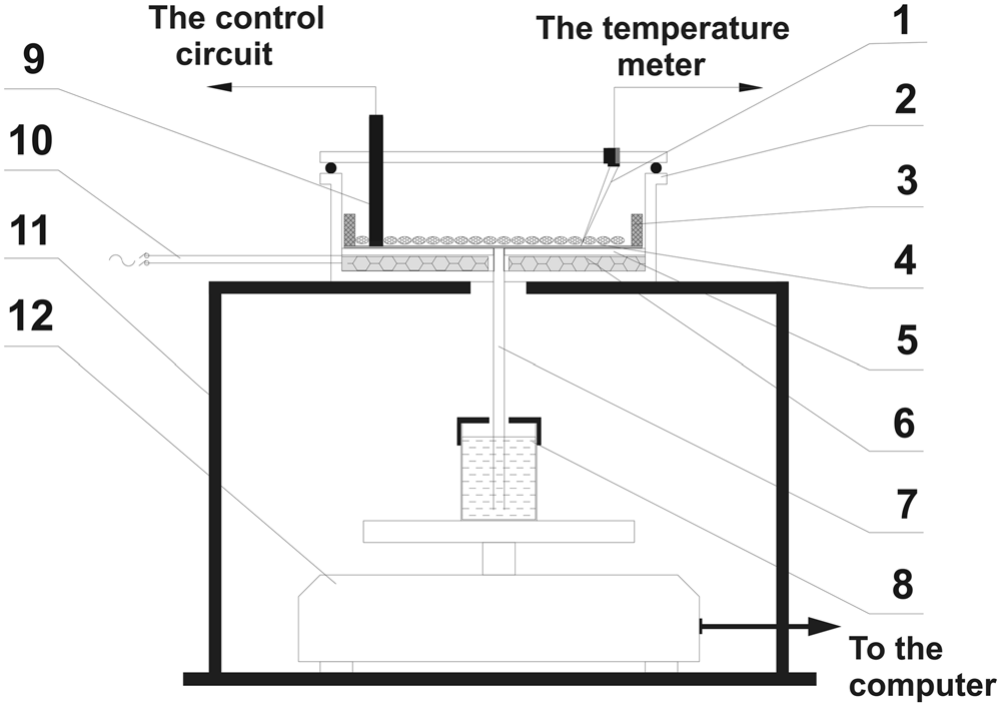
Schematic representation of the measurement system: 1 - thermocouple, 2 - thermostatic container for grains, 3 - mold for seeds, 4 - seeds, 5 - blotting paper fixed to the mold, 6 - heater, 7 - capillary, 8 - water container, 9 - Pt -100 sensor, 10 - heaters power supply, 11 - table, 12 - electronic scale.

Seed humidity was determined by the capillary-weight method in the water-intake system shown on Fig. 6. In the capillary-weight method the change in the weight of water in the beaker (with immersed capillary) standing on a scale is recorded. Seeds were placed in a sealed thermostatic container (2), where a constant temperature was provided by the heater supply regulator (6) co-operating with the Pt-100 sensor (9). The blotting paper (5) placed in the bottom of the container was closely attached to the glass capillary (7) pasted into the bottom of the vessel where the temperature was measured with a thermocouple (1). The other end of the capillary was placed in a water container (8) resting on the electronic scale (12) connected to the computer converging the measurement data. Measurements were carried out at 20 °C^74^. The change of water content in the sample ΔZ_K_ for the k-th measurement at a given time t was calculated from the formula:

1 $$\Delta {Z}_{K}=\frac{({m}_{PW}-{m}_{KW})-{m}_{PAR}}{{m}_{PZ}}\cdot 100 \% $$

where: *m*_*PW*_ - initial mass of water in the beaker at start of measurements, *m*_*KW*_ - mass of water in the beaker for k-th measurement, *m*_*PZ*_ - initial mass of grain at start of measurement, *m*_*PAR*_ - mass of water evaporating from the beaker through an annular hole in the cover: *Δm*_*PAR*_ = 5·10^−5^ kg·h^−1^.

Seed humidity was read from the graph presented in Fig. 2 which shows the dependence of water uptake for 500 amaranth seeds placed on a wet paper in a water-absorbing capillary-weight system for a given time. The chart shows the mean of the three measurement series. The seed moisture content increased gradually by 3–5%. The seed water uptake process lasted up to 24 hours until reaching a moisture content of 70%, just before the first sprouts appeared.

In the case of tests of seed germination, 100 pcs of seeds were placed on a single Petri dish. Five replicates have been done for each of the assigned humidity. Dry seeds, non-moisturized and untreated were used as controls. To study the content of photosynthetic pigments, 50 amaranth seeds were placed on Petri dishes. Experiments were conducted in triplicate for each seed moisture content. After 14 days from sowing, cotyledons for assigning of photosynthetic pigments were isolated.

The seeds were subjected to ELM stimulation when the assigned humidity was reached. The following stimulation variants were performed: L -stimulation with a He-Ne laser with a surface power density of 3 mW · cm^−2^ over 10 s, F - stimulation with an alternating magnetic field with frequency f = 50 Hz, magnetic induction B = 30 mT over 30 s, L + F - stimulation with both combined factors. The germination process was performed in the vegetation hall at 21 °C ± 2 ° in day/night conditions of 12/12 h, where artificial light of 500 lux intensity was used as an illumination. Laser stimulation doses were determined according to Gładyszewska^75^.

During the study, the seed germination kinetics was determined^31^. The percentage of germinated seeds *N*_*K*_ at a given time t was determined from the dependency below equation

2 $${N}_{K}=\frac{{n}_{K}}{{n}_{C}}\cdot 100 \% $$

where: *n*_*K*_ – number of germinated seeds at time *t*, *n*_*C*_ – total number of sown seeds. *N*_*K MAX*_ represents germination capacity. Germination rate (speed) *S*_*K*_ was determined from the equation:

3 $${S}_{K}=\frac{\Delta {n}_{K}}{\Delta {t}_{K}}$$

where: *Δn*_*K*_ – number of seeds assigned for a certain time interval *Δt*_*K*_, *Δt*_*K*_ – time interval between two neighboring germination counts. Relative germination index *W*_*K*_ was determined from the equation:

4 $${W}_{K}=\frac{n(t)}{{n}_{control}}$$

where: *n*(*t*) – number of seeds which germinated during time *t* for the stimulated variant, *n*_*control*_ – number of germinated seeds during time *t* for control sample.

The germination rate *S*_*K MAX*_, as well as *W*_*K MAX*_ reach the maximum values which can be read from the *S*_*K*_ = *f* (*t*) chart (not presented).

Chlorophylls and carotenoids were isolated from seedlings in total darkness with usage of acetone. In order to protect the sample against oxidation 0.5% of BHT (butylated hydroxy toluene) was added. UV-Vis spectra of acetone solutions of pigments were registered by means of double beam spectrophotometer Carry Bio. Concentrations of photosynthetic pigments were measured according to the procedure of Lichtenthaler and Buschmann^64^.

The results were analysed statistically based on Fisher’s ANOVA (NIR) test using STATISTICA 13.0 software. The analysis was carried out at the significance level α = 0.05 for two cases - the effect of seed stimulation compared to the control sample (table rows 1–5) and the influence of the change in seed humidity depending on the initial moisture content (columns 1 to 5). The different lower case letters (a and b) in the rows of Tables 1–5 indicate the existence of statistical differences between control (denoted as a lower case) and seed stimulating factors for a given level of humidity during stimulation. The uppercase letters (A and B) refer to the existence of statistical differences in the columns for the variant of the experiment (applied ELM stimulation).

## Data Availability

The datasets generated during and/or analyzed during the current study are available from the corresponding author on reasonable request.
